# Analysis of the Lockdown Effects on the Economy, Environment, and COVID-19 Spread: Lesson Learnt from a Global Pandemic in 2020

**DOI:** 10.3390/ijerph191912868

**Published:** 2022-10-08

**Authors:** Woraphon Yamaka, Siritaya Lomwanawong, Darin Magel, Paravee Maneejuk

**Affiliations:** 1 Center of Excellence in Econometrics, Chiang Mai University, Chiang Mai 50200, Thailand; 2 Faculty of Economics, Chiang Mai University, Chiang Mai 50200, Thailand

**Keywords:** COVID-19, economy, environment, lockdown, spatial spillover, nonlinear impact

## Abstract

Lockdown policies have been implemented to reduce COVID-19 transmission worldwide. However, the shutdown of activities has resulted in large economic losses, and it has been widely reported that lockdown measures have resulted in improved air quality. Therefore, many previous studies have attempted to investigate the impacts of the COVID-19-induced lockdowns on the economy, environment, and COVID-19 spread. Nevertheless, the heterogeneity among countries worldwide in the economic, environmental, and public health aspects and the spatial effects of decomposition have not been well investigated in the existing related literature. In this study, based on the cross-sectional data of 158 countries in 2020 and the proposed nonlinear simultaneous spatial econometric models, we investigate the nonlinear and spatial impacts of the COVID-19-induced lockdowns on the economy, environment, and COVID-19 spread. The findings show that lockdowns have had statistically significant negative economic impacts and beneficial environmental consequences but no effect on COVID-19 spread. Noteworthily, this study also found the length of lockdown periods to affect the three domains of interest differently, with a piece of empirical evidence that the imposition of lockdowns for more than 31 days a year could result in economic impairments but contribute to environmental improvements. Lockdowns were shown to have substantially reduced PM2.5 not only in the countries that imposed the measures but also indirectly in the neighboring countries as a spatial spillover effect.

## 1. Introduction

After COVID-19 was declared a global pandemic in March 2020, the governments of many countries imposed numerous measures to control the disease’s spread. One of the important measures was the area shut down or lockdown to limit the movement and activities of the population, as distancing between people can help reduce the spread of infection [1]. Ghosal, Bhattacharyya, and Majumder [2] examined the impact of lockdowns on COVID-19 spread in India and found a significant 61% reduction in infection rates one week after this measure was declared. Lockdowns have been found to help reduce the spread of infection and to be more effective if they are implemented at the onset of the disease outbreak [3]. 

However, lockdowns, when adopted, might cause enormous cumulative social and economic costs because, compared to SARS in 2003 and MERS in 2012, COVID-19 is much more infectious and fatal [4]. An economic impact assessment of COVID-19 by the International Monetary Fund (IMF) found that global GDP declined by more than USD 400 billion from the pre-pandemic level [5]. Furthermore, Mofijur et al. [6] found a significant adverse effect of COVID-19 on economic growth because the stringent interventions to restrict people’s movements and some economic activities had resulted in an immense economic loss for business firms, product manufacturers, and the service industry. The tourism economy, in particular, was shown to have been directly and heavily hit by the implementation of the lockdown measures. 

Although lockdowns have been shown to bring the economy to a halt, they can give time for degraded environmental and natural resources to recuperate and restore their health. For example, the decreased anthropogenic activities due to restrictions on travel and human/industrial activities resulted in reduced global carbon dioxide emissions [7]. Moreover, the International Energy Agency (IEA) disclosed that the continuing spread of COVID-19 lowered global energy consumption by more than 5–6% in 2020 relative to its level a year earlier. The remarkable fall in energy consumption due to lockdowns that limited or prohibited some industrial, trade and economic activities thus contributed to decreasing global CO_2_ emissions during the COVID-19 pandemic.

**Figure 1:**
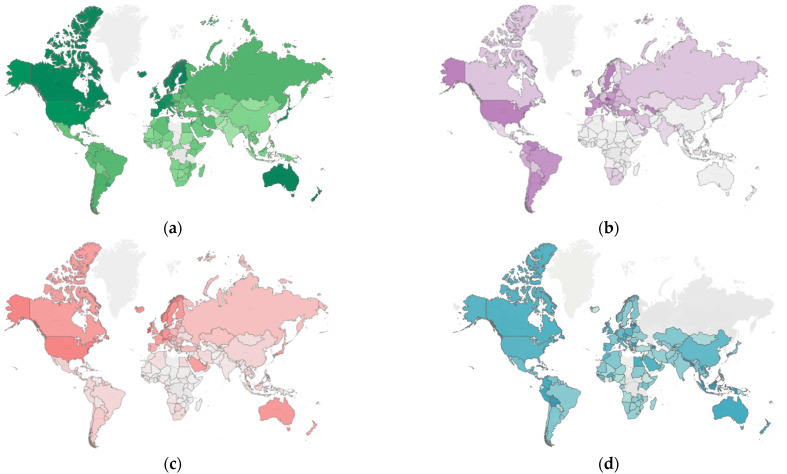
(**a**) Air quality (PM2.5), (**b**) the total number of COVID-19 cases per 100,000 people, (**c**) GDP per capita, and (**d**) the total days of lockdown of 158 countries worldwide in 2020. Note: The darker (lighter) the color, the higher (lower) the value of the indicator.

As mentioned above, the impact of lockdowns to reduce COVID-19 infection and mortality on the economy and the environment might have some significant spatial correlation. To provide an empirical piece of evidence of this spatial correlation, this study ventured into developing spatial maps showing the spatial distributions of air quality, the total number of COVID-19 cases, economic development, and cumulative COVID-19 cases in 2020, as shown in Figure 1. The degree of intensity is represented by the color tone, where the higher (lower) degree of intensity corresponds to the darker (lighter) color.

Figure 1 above displays the presence of spatial correlations between lockdowns, environment, and COVID-19 infections at one locality and those at neighboring localities, which are further discussed below. 

The first issue involves the significance of the geographic position of each country. We can observe that the levels of GDP, air quality (represented by PM2.5 concentration), and COVID-19 infections are characteristically similar among countries located adjacently or nearby that can be easily visualized as a cluster. Therefore, the spatial analysis of lockdown effects should take into account the factor of the distance between countries, particularly considering that air pollutants and contagious diseases are highly mobile. Consequently, a country imposing lockdown measures can produce impacts on the economy, environment, and public health of its neighboring countries and perhaps beyond, known as spatial spillovers. The external impact can be positive and negative. For example, on the one hand, a lockdown in a country not only results in a GDP fall locally but also can shrink the size of border trade, negatively affecting the GDP of the neighboring countries that are its trading partners. On the other hand, the preventive restrictions in a country that contribute to improved air quality locally might help lower pollution concentrations in surrounding countries [1]. Sajadi et al. [8] added that COVID-19 was transmitted from one country to its immediate neighboring countries before rolling out to more distant countries and that the geographic and climatic differences across countries can be a factor driving or discouraging the disease transmission, as they found the COVID-19 outbreak to spread from the source to countries of the Eastern World first and then to Western countries later in a narrow band in which the affected countries had similar weather patterns. Figure 1 also reveals the zonal patterns of countries or regions in Europe and North America having comparable levels of COVID-19 infections in 2020. Paulo [9] and Yang, Li, and Cao [10] studied whether the geographical location and climate can affect disease spread and found both factors to be significant in influencing the transmission of COVID-19, with the speed of infection spread in cool-climate communities or regions higher than in hot-climate areas. Thus, the present investigators believe it is crucial to consider the geographical factors and spatial effects discussed in our study. From a literature review, we came across the studies by Xiong et al. [11], Hasraddin [12], and Franch-Pardo et al. [13] that addressed the spatial analysis of COVID-19. However, we found no works that had been performed on the use of the spatial concept for analyzing the impacts of COVID-19 lockdowns in one country or group of countries on the others regarding the economy, environment, and infections. The only work with a similar feature is that of Bourdin et al. [14], using the spatial relationship concept to analyze the effects of lockdowns on the disease spread and concentration in Italy, with one of their findings indicating that the COVID-19 severity in cities or towns highly connected to the external world was higher than elsewhere in the country.

The second point addresses the heterogeneity among countries worldwide in the economic, environmental, and public health aspects. Thus, with heterogeneity, COVID-19 lockdowns should bring about impacts on these three domains differently across countries. Previous studies, mostly focusing on the lockdown impacts on the individual country, already lend support to our belief because their findings as a whole suggest the impacts can be different in magnitude and direction in different countries or regions [2]. Countries with long lockdown applications reduced relatively more COVID-19 cases compared to countries with short lockdowns [15]. Gatto et al. [16] maintain that lockdown programs can reduce transmission dynamics of COVID-19 by about 45%. Instead, Tobías [17] reported with a graphical illustration that after the first lockdown in Spain and Italy, the slopes of daily confirmed cases were flattened. However, the slopes became steeper in the second lockdown. From an environmental point of view, the nonlinear impact of COVID-19 spread on the environment was recently confirmed by Wang and Li [7]. They revealed that the impact of lockdowns on pollution was heterogeneous. The response of air pollutants to lockdowns was different across cities (Wuhan, Milan, London, Mexico, New York City, Bandra, Madrid, and Tokyo). From an economic perspective, lockdowns have been shown to harm the economy (Ali et al. [18]) following the disruption of various economic and industrial activities. However, the short application of a lockdown was reckoned to be not enough to restrain the pandemic and could result in more economic damage in the long term if the pandemic is prolonged. Therefore, an intense and long application of lockdowns is generally required despite the risk of deepening economic hardship [1]. Coccia [15] also showed that lockdowns have different effects on the economy depending on the number of lockdown days, such as a small impact from a short period of lockdown and a large effect from a lengthy lockdown. The inconsistent conclusions from previous studies motivated us to use nonlinear econometric models to better capture the different impacts of lockdowns on the economy, environment, and public health, taking into account spatial heterogeneity. Importantly, all previous models analyzing the impacts of lockdowns on these three domains of concern and interest failed to consider the potentially nonlinear effects. In this respect, we are convinced our integration of nonlinear and spatial effects of COVID-19 lockdowns into an econometric modeling framework for analysis will provide many useful results and stylized facts for policymakers to consider our policy recommendations.

As indicated earlier, lockdowns imposed primarily to curb COVID-19 contagion and mortality can also have both beneficial and undesirable economic and environmental consequences depending on the considered variables. Governments that have implemented lockdowns are now facing a crucial challenge concerning the trade-off in part or as a whole between different attainable goals, and in the present context, there is a trade-off to consider between the gain and the loss in the three domains. In this connection, the policy decision to balance the attainment of economic, environmental, and public health goals should be given a high priority. Consequently, the main aim of the present study was to determine the impacts of lockdowns on the economy, environment, and COVID-19 spread at the global scale under our conviction that the impacts are nonlinear across countries due to spatial factors and relations. Furthermore, this study estimated the possible linkages between economy, environment, and COVID-19 spread. Thus, the simultaneous equation model was considered to construct our empirical model. Note that this model accounts for likely correlations among the individual behavioral equations for the economy, environment, and COVID-19 infections. Precisely, it allowed us to identify the underlying nature of the simultaneous relationships among these three endogenous variables and also deal with the endogeneity problem [19], which generally occurs in a single equation model. To this end, we suggested using the simultaneous spatial kink equations model to establish empirically and comprehensively the nonlinear and spatial effects of lockdown measures such that we could use our findings to draw appropriate policy recommendations for achieving the economic, environmental, and public health goals. To the best of our knowledge, our work is the first attempt to extend the simultaneous spatial equations model into the nonlinear framework. This system model allowed us to investigate the direct and indirect nonlinear influence of each regressor and spatial variable on the dependent variable, which also reflects the value and innovation of this paper.

The remainder of this paper is organized as follows. Section 2 presents the literature review. Section 3 presents the data and the proposed models used in this analysis. Section 4 provides the analysis of empirical results, Section 5 is the discussion of the results, and Section 6 gives conclusions and Section 7 provides recommendations.

## 2. Literature Review

### 2.1. Impact of COVID-19-Induced Lockdowns on the Environment

COVID-19 lockdowns reduced the extent of economic and transportation activities after state or local governments ordered the closing of some business and industrial operations. The reduced economic activities and traffic led to declines in energy and fuel consumption and, eventually, the remarkable restoration of environmental quality. Hashim et al. [20] undertook a study comparing the PM2.5 and O_3_ concentrations between before- and after-lockdown periods in Bagdad, Iraq, and concluded that lockdown measures could reduce the PM2.5 concentrations by 6% and increase O_3_ levels by 13%, indicating the improvement of air quality. Their main finding is consistent with the study results of Mahato, Pal, and Ghosh [21], showing that lockdowns could significantly reduce PM2.5 concentration in Delhi, India, by 39% relative to the pre-COVID period, contributing to a considerable improvement in air quality. In the same line, Fu, Purvis-Roberts, and Williams [22] assessed the impact of the COVID-19 pandemic lockdowns on air quality in 20 major cities around the world. They found that the lockdown measures led to lower air pollution in many megacities due to the reduced anthropogenic activities and that the reduction of air pollutants was also a matter of pre-established environmental regulations and local climatic characteristics, as they traced a substantial reduction in air pollutants in the Asian region to prevailing stringent pollution-control policies and the monsoon climate. Mandal and Pal [23] also observed lockdown contributions to reduced land traffic and transportation activities, which in turn helped lower PM2.5 concentrations. The lockdown benefit to air quality has been empirically established for many countries such as Spain and Italy [4], India [21,24], Brazil [25], and China [26]. However, a study of greater depth can reveal more clearly the varying air pollution reduction levels across countries, as suggested by Roy et al. [27], who found from their geospatial analysis of the Asian region that Katmandu, Jakarta, and Hanoi experienced the highest reductions in air pollution compared to other cities, by 40–47%, as a result of the COVID-19 induced lockdowns. 

### 2.2. Impacts of COVID-19 Induced Lockdowns on the Economy

Caselli et al. [5], Mofijur et al. [6], and Ali et al. [18] explored and found statistically significant economic effects of lockdowns during the COVID-19 pandemic in many countries around the world. The severe impacts of lockdowns on certain social groups were obvious from the reduced income levels, rising unemployment rates, worker layoffs, increased household debt, and the overall economic recession. However, lockdowns are believed to be the most effective in the public health interest considering the pandemic outcomes in non-lockdown countries such as Sweden that favored voluntary social distancing, permitted gatherings of no more than 50 persons, and imposed no remote monitoring of employees to protect individual’s privacy in comparison to outcomes in full-lockdown countries such as China, which mandated the lockdowns of cities, regions, localities, and public transportation [2]. Lockdowns resulted in lower incomes for many businesses and shops, as well as the impossibility for some businesses to continue their operations, which in turn resulted in sizable employee layoffs [1]. However, Caselli, Grigoli, and Sandri [28] found that lockdowns, if implemented immediately after an outbreak, could concretely and effectively curb the COVID-19 spread, with some small and short-term damage to economic health that could be easily restored to a state of normalcy. However, lifting lockdown measures when the pandemic remained highly prevalent could not help the economy to recover immediately and sustainably.

### 2.3. Impact of COVID-19-Induced Lockdowns on the Number of COVID-19 Infections 

While countries with advanced healthcare systems such as Italy, the USA, Spain, and France failed to retard the COVID-19 contagion due to their lack of social distancing restrictions, India, with the world’s second-largest population and an undeveloped healthcare system, has been successful in controlling the disease spread considering its lower rates of infections and deaths because the Indian population observed the stringent social distancing requirements [29]. Lockdowns, with their social distancing and isolation implications, if imposed adequately and timely, will help reduce the disease spread and the number of infections (International Monetary Fund, 2020). However, the number of COVID-19 infections in a locality was found to be correlated more with the local population density [30]. Ghosal, Bhattacharyya, and Majumder [2] noted that although lockdowns could reduce the number of infections by 61% globally, the reduction was faster in countries that imposed the measures in the early phase of the outbreak, such as Belgium, Austria, New Zealand, India, and Hungary, than in countries instituting lockdowns in the later phase, such as Spain, Germany, Italy, the United Kingdom, and France. Studies by Atalan [1] and Caselli, Grigoli, and Sandri [28] revealed similar findings that the COVID-19 pandemic caused a negative economic impact worldwide because the lockdown measures to limit transportation and human movements not only brought about a halt to other related economic activities but also helped reduce fuel consumption and air pollution. However, Renardy et al. [31] provided a different view, stating that lockdowns might not reduce confirmed cases but only delay them. Similarly, Farsalinos et al. [32] cautioned that lockdowns might work well only in the short term by delaying the possibility of virus transmission, and horizontal lockdowns cannot be used as the core of a long-term strategy for pandemic mitigation because of important adverse social, economic and health effects that have yet to be fully elucidated. The recent work of Coccia [15] compared the average confirmed cases in the countries applying long and short lockdowns. The results showed that average confirmed cases do not suggest a difference in the evolution of the COVID-19 pandemic in countries with a longer or a shorter duration of national lockdowns.

### 2.4. Simultaneous Relationships among COVID-19 Spread, Economy, Environment

Many scholars have studied the relationships between COVID-19 transmission, the economy, and the environment. The interactions between these variables are widely recognized in the literature [1,6,18,33,34]. These studies revealed that COVID-19 produced an adverse impact on human life, the economy, and the environment. Mofijur et al. [6] and Nundy et al. [34] confirmed that COVID-19 can, on the one hand, reduce economic activity and lower economic growth, and on the other hand, reduce environmental pollution. However, some recent studies have shown that environmental and socio-economic factors could intensify the spread of the COVID-19 virus [33,35]. Ahmed et al. [33] analyzed this considering 70 cities/provinces of different countries around the world. They found that countries with a higher level of economic development were more likely to have a lower number of COVID-19 infected cases than otherwise. This is quite reasonable, as the advanced economies tend to have a larger number of social events, and their people spend more time traveling and joining these social events, making it easier for virus diffusion. Meanwhile, rich or poor countries with heavier air pollution were more likely to have more COVID-19 cases. Bourdrel et al. [35] explained that air pollution could provoke the COVID-19 pandemic in two ways. (1) Pollution could reduce the level of immunity in humans, making people more vulnerable to COVID-19 infection. (2) COVID-19 disease can be transmitted through microscopic particles or combined with ultrafine aerosols. Thus, COVID-19 becomes easier to spread in countries with a higher concentration of air pollutants PM2.5).

The linkage between economic development and environmental degradation has always been a hot issue in the study of environmental economics. One of the famous theories explaining this relationship can be referred to as the Environmental Kuznets Curve (EKC) hypothesis. This theory postulated an inverted-U-shaped relationship between these two variables, i.e., the environmental pressure increases up to a certain level as income goes up; after that, it decreases [36]. This nonlinear relationship between these two variables has already been confirmed by Maneejuk et al. [37]. The environment–growth nexus has mainly been examined with respect to the following two competing hypotheses: the unidirectional hypothesis and the bidirectional hypothesis. The unidirectional hypothesis (either from the environment to economic growth or from economic growth to the environment) and the bidirectional hypothesis have already been confirmed by Govindaraju and Tang [38], Wang [39], Vo, Vo, and Le [40], and Nasreen et al. [41]. 

The above-mentioned perspectives are interrelated, and these facts motivated us to investigate the nexus among these three variables through the use of the simultaneous spatial kink equations models simultaneously. These models are appropriate to handle not only the integrated analytical framework discussed above but also the nonlinear and spatial effects. Additionally, the simultaneous equation techniques are capable of solving some technical and econometric issues (such as endogeneity problems and correlated error issues) associated with the use of a single equation in estimating the links between COVID-19 spread, economy, as well as the environment [42].

## 3. Data and Methodology

### 3.1. Data

The main property of our suggested model is that it allows for endogenous variables to be incorporated as explanatory variables in other equations. Further, each equation contains several specific factors (exogenous variables). In this study, we used cross-sectional data covering 158 countries (48 Asian, 47 African, 23 American, and 40 European countries, see Table A1) in 2020 to determine the impacts of the COVID-19-induced lockdowns on the economy, environment, and COVID-19 spread. For the estimation by our simultaneous spatial models, we considered GDP per capita (*GDP*), PM2.5 (*PM*), and the number of COVID-19 confirmed cases (COVID), which are indicators of economic growth, environmental quality, and COVID-19 spread, as endogenous variables, and the following as exogenous variables, including population density (Pop), COVID-19 Recovery Index (*Index*), trade openness (*Open*), foreign direct investment (*FDI*), real interest rate (*r*), and temperature (*Temp*). Then, to achieve the goal of our study, we used the following three equations, which were modeled simultaneously.

(1) $$\begin{array}{cc} \ln GDP_{i} & =f{\left(\ln Lock_{i},\ln PM_{i},\ln COVID_{i},\ln Pop_{i},\ln Index_{i},\ln Open_{i},\ln FDI_{i},\ln r\right)}_{i}\\ \ln PM_{i} & =f(\ln Lock_{i},\ln COVID_{i},\ln GDP_{i},\ln Pop_{i},\ln Open_{i},\ln Temp_{i})\\ \ln COVID_{i} & =f(\ln Lock_{i},\ln GDP_{i},\ln PM_{i},\ln Pop_{i},\ln Index_{i}) \end{array}$$

The system of Equation (1) presents the three-way linkage between ln*GDP*, ln*PM*, and ln*COVID* in which ln*Lock* plays a vital role affecting them. The choice of the variables for this investigation was made based upon the availability of data and the literature review. Population density (ln*Pop*) is another key variable existing in all equations in the system, as it has been proved to have a positive impact on economic growth, environment, and the spread of COVID-19 [7,33,43,44]. Trade openness (ln*Open*) is also added as the determinant of economic development [45] and air pollution [46]. Moreover, there is an impressive body of literature looking at the influence of patient recovery rate on economy and COVID-19 spread [47,48]. We thus also considered ln*Index* in the GDP and COVID-19 equations. With respect to ln*FDI* and ln*r*, these variables exert a theoretical effect on economic growth [49,50]. Finally, in terms of temperature, ln*Temp* was added as the determinant of air pollution [33]. The descriptive statistics of these variables are presented in Table 1.

### 3.2. Methodology

#### 3.2.1. Spatial Autocorrelation 

We consider the spatial autocorrelation concept to examine the presence of spatial correlations between variables at one locality and variables at neighboring localities. Specifically, this method is used to analyze whether the variable of interest is influenced by the spatially dependent conditions. In this study, we use the Moran’s I index to measure spatial autocorrelation.

(2) $$I=\frac{n\sum_{i=1}^{n}{\sum_{j=1}^{n}w}_{ij}\left(x_{i}-\bar{x}\right)\left(x_{j}-\bar{x}\right)}{\sum_{i=1}^{n}{\left(x_{i}-\bar{x}\right)}^{2}\sum_{i=1}^{n}\sum_{j=1}^{n}w_{ij}},$$

where $x_{i}$ and $x_{j}$ are the variables of country i and j, respectively, $\bar{x}$ is the average of the observed values, n is the number of countries, $w_{ij}$ is the matrix n×n spatial weight of countries i and j ($w_{ij}=1$, if country i is a neighbor to j; else $w_{ij}=0$ country i is not a neighbor to j). The value of Moran’s I index ranges between −1 and 1. I>0 indicates the positive spatial correlation, I<0 indicates the negative spatial correlation, and I=0 indicates no spatial correlation, and it is randomly distributed in the space [51].

#### 3.2.2. Simultaneous Spatial Kink Equations

As the countries in a neighborhood may emulate each other, there might exist a spatial dependence [52]. Nguyen et al. [53] mentioned in their work that the assumptions of no correlation between independent variables and errors and the expected value of the error term equals zero are invalid in the linear regression model if there exists spatial dependence. Therefore, we could not ignore the presence of spatial dependence in our econometric models. In this study, we are dealing with the simultaneous nonlinear relationships between economic development, environmental quality, and the COVID-19 pandemic, where each equation contains both endogenous and exogenous variables. Hence, we incorporate the spatial factors of LeSage [52] into the simultaneous kink equation model of Maneejuk, Pastpipatkul, Sriboonchitta [54] and Maneejuk, Yamaka, and Sriboonchitta [55]. According to LeSage [52] and LeSage and Pace [56], there are three forms of spatial models, namely the spatial autoregressive model (SAR), spatial error model (SEM), and spatial Durbin model (SDM). We thus consider these three forms of simultaneous spatial kink equation models as follows.

1. Simultaneous spatial autoregressive kink equations (SSAKE)

This study considers the two-regime model, which takes the form as

(3) $$\begin{array}{l} y_{1}=\alpha_{1}+\rho_{1}\sum_{j=1}^{n}w_{ij}y_{1}+\sum_{g=1}^{G1}\beta_{1,g}^{-}{\left(Y_{1}-\gamma_{1,g}\right)}_{-}+\sum_{g=1}^{G1}\beta_{1,g}^{+}{\left(Y_{1}-\gamma_{1,g}\right)}_{+}+\sum_{k=1}^{K1}\beta_{1,G1+k}^{-}{\left(X_{1,k}-\gamma_{1,G1+k}\right)}_{-}+\sum_{k=1}^{K1}\beta_{1,G1+k}^{+}{\left(X_{1,k}-\gamma_{1,G1+k}\right)}_{+}+\sum_{q=1}^{Q}\delta_{1,q}Z_{1,q}+\varepsilon_{1}\\ \vdots \\ y_{m}=\alpha_{m}+\rho_{m}\sum_{j=1}^{n}w_{ij}y_{m}+\sum_{g=1}^{Gm}\beta_{m,g}^{-}{\left(Y_{m}-\gamma_{m,g}\right)}_{-}+\sum_{g=1}^{Gm}\beta_{m,g}^{+}{\left(Y_{m}-\gamma_{m,g}\right)}_{+}+\sum_{k=1}^{Km}\beta_{m,Gm+k}^{-}{\left(X_{m,k}-\gamma_{1,Gm+k}\right)}_{-}+\sum_{k=1}^{Km}\beta_{1,Gm+k}^{+}{\left(X_{m,k}-\gamma_{1,Gm+k}\right)}_{+}+\sum_{q=1}^{Q}\psi_{m,q}\sum_{j=1}^{n}w_{ij}Z_{m,q}+\varepsilon_{m} \end{array}$$

where $y_{h}$ and $\varepsilon_{h}$ are vectors of n×1 dependent variable and error of equation h. $Y_{h}$ is an n×Gh matrix of regime-dependent endogenous variable in equation h, and $X_{h,k}$ is vector of n×1 regime-dependent exogenous variable k of equation h, whereas h=1,...,m. Note that $Y_{h}$ and $X_{h,k}$ are the independent variables, which have a nonlinear impact on $y_{h}$. $Z_{h,q}$ is an exogenous variable of equation h, which has a linear relationship with $y_{h}$. In this model form, we incorporate $\rho_{h}\sum_{j=1}^{n}w_{ij}y_{h}$ (spatial lag term) to control the spatial dependence of the dependent variable in equation h. Note that $\sum_{j=1}^{n}w_{ij}y_{h}$ is typically referred to as the spatial lag of $y_{h}$.

Additionally, as we are focusing on the nonlinear relationship structure in our models, we apply the indicator function of Hansen [57], ${\left(a\right)}_{-}=\min\left(a,0\right)$ and ${\left(a\right)}_{+}=\max\left(a,0\right)$, to separate regime-dependent endogenous and regime-dependent exogenous variables into two regimes, namely X,Y≤γ and X,Y>γ, respectively (where a is X−γ or Y−γ). γ is the kink parameter or kink point. $\beta_{}^{-}$, $\beta^{+}$ and δ are the estimated coefficients of the system equations, and $\rho_{h}$ represents the spatial autoregression coefficient in equation h. In this system equations model, each equation is linked to the others by the covariance structure of the errors $E(\varepsilon \varepsilon^{\prime })=\Sigma$ where $\varepsilon =\left(\varepsilon_{1},...,\varepsilon_{m}\right)$. The error structure is then assumed to follow the multivariate normal distribution N(E(ε)=0,Σ) [53].

2. Simultaneous spatial error kink equations (SSEKE)

In this form, the model only includes the spatial factor in the error term, and we can write its formula as

(4) $$\begin{array}{l} y_{1}=\alpha_{1}+\sum_{g=1}^{G1}\beta_{1,g}^{-}{\left(Y_{1}-\gamma_{1,g}\right)}_{-}+\sum_{g=1}^{G1}\beta_{1,g}^{+}{\left(Y_{1}-\gamma_{1,g}\right)}_{+}+\sum_{k=1}^{K1}\beta_{1,G1+k}^{-}{\left(X_{1,k}-\gamma_{1,G1+k}\right)}_{-}+\sum_{k=1}^{K1}\beta_{1,G1+k}^{+}{\left(X_{1,k}-\gamma_{1,G1+k}\right)}_{+}+\sum_{q=1}^{Q}\delta_{1,q}Z_{1,q}+\lambda_{1}\sum_{j=1}^{N}w_{ij}\mu_{1}+\varepsilon_{1}\\ \vdots \\ y_{m}=\alpha_{m}+\sum_{g=1}^{Gm}\beta_{m,g}^{-}{\left(Y_{m}-\gamma_{m,g}\right)}_{-}+\sum_{g=1}^{Gm}\beta_{m,g}^{+}{\left(Y_{m}-\gamma_{m,g}\right)}_{+}+\sum_{k=1}^{Km}\beta_{m,Gm+k}^{-}{\left(X_{m,k}-\gamma_{1,Gm+k}\right)}_{-}+\sum_{k=1}^{Km}\beta_{1,Gm+k}^{+}{\left(X_{m,k}-\gamma_{1,Gm+k}\right)}_{+}+\sum_{q=1}^{Q}\delta_{m,q}\sum_{j=1}^{n}w_{ij}Z_{m,q}+\lambda_{m}\sum_{j=1}^{N}w_{ij}\mu_{m}+\varepsilon_{m} \end{array}$$

where $\lambda_{h}$ is the coefficient of the spatial error term $\sum_{j=1}^{N}w_{ij}\mu_{m}$ of equation h.

3. Simultaneous spatial Durbin kink equations (SSDKE)

In the third form, we consider the spatial correlation of both exogenous and endogenous variables in the model to be expressed as

(5) $$\begin{array}{l} y_{1}=\alpha_{1}+\rho_{1}\sum_{j=1}^{n}w_{ij}y_{1}+\sum_{g=1}^{G1}\beta_{1,g}^{-}{\left(Y_{1}-\gamma_{1,g}\right)}_{-}+\sum_{g=1}^{G1}\beta_{1,g}^{+}{\left(Y_{1}-\gamma_{1,g}\right)}_{+}+\sum_{k=1}^{K1}\beta_{1,G1+k}^{-}{\left(X_{1,k}-\gamma_{1,G1+k}\right)}_{-}+\sum_{k=1}^{K1}\beta_{1,G1+k}^{+}{\left(X_{1,k}-\gamma_{1,G1+k}\right)}_{+}+\sum_{q=1}^{Q}\delta_{1,q}Z_{1,q}+\\ \;\;\;\;\;\;\;\;\;\;\sum_{g=1}^{G1}\theta_{1,g}^{-}{\left(\sum_{j=1}^{N}w_{ij}Y_{1}-\gamma_{1,g}\right)}_{-}+\sum_{g=1}^{G1}\theta_{1,g}^{+}{\left(\sum_{j=1}^{N}w_{ij}Y_{1}-\gamma_{1,g}\right)}_{+}+\sum_{k=1}^{K1}\theta_{1,G1+k}^{-}{\left(\sum_{j=1}^{N}w_{ij}X_{1,k}-\gamma_{1,G1+k}\right)}_{-}+\sum_{k=1}^{K1}\theta_{1,G1+k}^{+}{\left(\sum_{j=1}^{N}w_{ij}X_{1,k}-\gamma_{1,G1+k}\right)}_{+}+\sum_{q=1}^{Q}\psi_{1,q}\sum_{j=1}^{n}w_{ij}Z_{1,q}+\varepsilon_{1}\\ \vdots \\ y_{m}=\alpha_{m}+\rho_{m}\sum_{j=1}^{n}w_{ij}y_{m}+\sum_{g=1}^{Gm}\beta_{m,g}^{-}{\left(Y_{m}-\gamma_{m,g}\right)}_{-}+\sum_{g=1}^{Gm}\beta_{m,g}^{+}{\left(Y_{m}-\gamma_{m,g}\right)}_{+}+\sum_{k=1}^{Km}\beta_{m,Gm+k}^{-}{\left(X_{m,k}-\gamma_{m,Gm+k}\right)}_{-}+\sum_{k=1}^{Km}\beta_{m,Gm+k}^{+}{\left(X_{m,k}-\gamma_{m,Gm+k}\right)}_{+}+\sum_{q=1}^{Q}\delta_{m,q}Z_{m,q}+\\ \;\;\;\;\;\;\;\;\;\;\sum_{g=1}^{Gm}\theta_{m,g}^{-}{\left(\sum_{j=1}^{N}w_{ij}Y_{m}-\gamma_{m,g}\right)}_{-}+\sum_{g=1}^{Gm}\theta_{m,g}^{+}{\left(\sum_{j=1}^{N}w_{ij}Y_{m}-\gamma_{m,g}\right)}_{+}+\sum_{k=1}^{Km}\theta_{m,Gm+k}^{-}{\left(\sum_{j=1}^{N}w_{ij}X_{m,k}-\gamma_{m,Gm+k}\right)}_{-}+\sum_{k=1}^{Km}\theta_{m,Gm+k}^{+}{\left(\sum_{j=1}^{N}w_{ij}X_{m,k}-\gamma_{m,Gm+k}\right)}_{+}+\sum_{q=1}^{Q}\psi_{m,q}\sum_{j=1}^{n}w_{ij}Z_{m,q}+\varepsilon_{m} \end{array}$$

where $w_{ij}y_{h}$, $\sum_{j=1}^{n}w_{ij}Y_{h}$,$\sum_{j=1}^{n}w_{ij}X_{k,h}$ and $\sum_{j=1}^{N}w_{ij}Z_{h.q}$ are the spatial lag of endogenous and exogenous variables on the right-hand side of the equation. In this case, we reduce the complexity of our model by assuming that the kink point of each endogenous and spatial lag endogenous variable is the same. This assumption is also held for exogenous and spatial lag exogenous variables. $\theta^{-},\theta^{+},\psi$ are the spatial error coefficients. 

To estimate all of the unknown parameters in these three models, we use the maximum likelihood estimator. All parameters are estimated by maximizing the likelihood of the model with respect to each unknown parameter. Note that the multivariate normal likelihood is assumed in this study.

In the case of the SSDKE model, the parameters $\theta^{-},\theta^{+},\psi$ cannot be used to explain the influence of spatial lag independent variables on the dependent variable, as they contain some bias. Thus, we apply the partial differentiation method of LeSage and Pace [56] to decompose the direct and indirect (spatial spillover) effects of the independent variables on the dependent variable.

## 4. Results

### 4.1. Spatial Autocorrelation Test

The first part of this study involves the spatial correlation test using Moran’s I technique. We divide the data into four sub-regions (Asia, Europe, Africa, and America) and examine the spatial correlation between a variable in one country and the same variable in neighboring countries in each sub-region and the world. In essence, all variables are tested to confirm the presence of their spatial effects. The results as presented in Table 2 show that all variables are statistically significant and have Moran’s I values greater than 0, indicating the existence of positive spatial influence in all regions as well as the world. In other words, the results indicate that the data sets of each variable of countries located nearby or next door tend to move in the same direction. Thus, the test results support the suitability of including spatial dependence in the econometric models. 

However, the degree of spatial dependence differs across the three domains of interest, as Moran’s I values of the economy for all regions are stronger than those of the environment and COVID-19 spread. These results strongly imply that the spatial economic effects of the 158 countries are less random and scattered than the effects of spatial environment and COVID-19 spread.

### 4.2. Kink Effect Test

Before estimating the three proposed econometric models, we tested whether each independent variable has a nonlinear relationship with dependent variables in each model equation. Such kink effect was tested using the likelihood ratio (LR) with the null hypothesis that $H_{0}:\beta^{-}=\beta^{+}$ (linear relationship) against the alternative hypothesis that $H_{0}:\beta^{-}\neq \beta^{+}$ (nonlinear relationship); and the results are shown in Table 3.

Table 3 presents the test results on whether the nonlinear relationship exists in the pair of an independent and dependent variable; or, in other words, whether a kink point presents in their relationship. The figure shown for a variable pair is the F-statistic value for the test of the kink effect, and the number below it is the estimated kink point value for the pair having a kink effect. The LR test was performed for the spatial regression model in three forms, including spatial lag, spatial error, and spatial Durbin. Similarly significant findings were obtained from the tests for the three different models: (1) for the economic equation, ln*GDP* has the nonlinear relationship with ln*Lock*, ln*PM*, ln*COVID* ln*Pop* and ln*Index*; (2) for the air quality equation, ln*PM* obtains the nonlinear influence from ln*Lock*, ln*COVID* and ln*GDP*; and (3) for the COVID-19 spread equation, ln*COVID* has the nonlinear relationship only with ln*GDP* and ln*Index*. 

### 4.3. Comparison of Model Performance

The following analysis was to help determine the optimal spatial model for estimation using our acquired data sets. To compare model performance, we estimated the spatial models for the economy, environment, and COVID-19 in both linear and nonlinear forms. Furthermore, we fitted two non-spatial models, namely the simultaneous kink equation (SKE) and simultaneous linear equation (SLE) systems, on the same data sets to verify the validity of the spatial autocorrelation assumption. The estimation results are reported in Table 4, and the optimal model is determined by the highest Loglikelihood value and the lowest Bayesian information criterion (BIC) value. Thus, the spatial Durbin model is the best model for extension into the simultaneous spatial Durbin kink equation model for the subsequent estimation and analysis of the impacts of COVID-19 lockdowns on the economy, environment, and COVID-19 infections.

### 4.4. Estimation Results from the Optimal Model

The analysis in Section 4.3 indicates the best model for fitting our data sets is in the form of simultaneous spatial Durbin kink equations (SSEKE), while the test results in Section 4.2 pinpoint the factors that can exert the nonlinear effect on the relationship between the dependent and independent variables. Therefore, we have well-defined information and frameworks for specifying an econometric model that can fit suitably on our data sets. The parameter estimates from our SSEKE model are presented in Table 5.

The estimation results in Table 5 reveal that the relationships between the independent variables and their corresponding dependent variables are different in degree of strength among the three equations but nonlinear in the main feature for all equations. Specifically, the effects of various explanatory variables on GDP, air quality, and COVID-19 infections are nonlinear, with the change in a relationship taking place at their respective kink points. With the nonlinear effect, the coefficient of an explanatory variable will take two different slopes (or impact strength) values for the portions before and after the kink point, symbolized by $\beta^{-}$ and $\beta^{+}$, respectively. Meanwhile, the variables that have a linear effect on the dependent variables will have a single coefficient value, as indicated by the symbol β.

Another feature of the present study is the inclusion in the analysis of the spatial effects captured by the parameter estimates, namely $\beta_{W\ln PM}^{+}$ and $\beta_{W\ln Lock}^{+}$ in the ln*GDP* equation, $\beta_{W\ln Lock}^{+}$ in the ln*PM* equation, and $\beta_{W\ln Lock}^{}$ in the ln*COVID* equation that express the magnitude of the spillover effect of a lockdown in one country/region on the economy, environment, and COVID-19 infections of its neighboring countries/regions. However, the findings from the SSEKE model cannot be used for direct interpretation of the spatial spillover effects because the spatial data pooled over time are influenced by the weighted spatial matrix [56]. Therefore, we have to derive the direct effect and indirect effect from the estimated total effect of each variable. 

### 4.5. Direct and Indirect Effects of Lockdowns

Table 6 and Table 7, respectively, present the calculated direct and indirect spatial spillover effects. From Table 6, we can observe that a lockdown has a statistically significant impact on most considered variables, as hypothesized in this study. The findings can be discussed from two theoretical perspectives, as follows. Firstly, among the three considered domains, a lockdown was found to have a nonlinear impact only on the environment represented by the PM2.5 concentrations and the economy represented by GDP. Given the kink point, ln*Lock* < 3.441 (the kink value as shown in Table 3) or lockdown of less than or equal to exp (3.441) = 31.218 days per year affected ln*GDP* negatively at the statistically significant level, while ln*Lock* > 3.441 or lockdown of more than 31.218 days had a significant negative effect on ln*GDP*. Our findings align with those reported by Atalan [1] and Caselli, Grigoli, and Sandri [28]. However, we wish to highlight an interesting observation about the different magnitudes of impact before and after the kink point; one more lockdown day beyond 31.218 days per year can impair the economy by 0.312%, while one less lockdown day than the 31.2181 days’ point will damage the economy by only 0.193%. Secondly, for lockdown impact on air quality, it was found that the lockdown days beyond the kink point, or ln*Lock* > 2.421 with kink at exp (2.421) = 11.257 days per year, had no effect on ln*PM*. However, if ln*Lock* < 2.421, a 1% increase in the number of lockdown days will lower the PM2.5 concentrations by 0.012%. We are convinced a lockdown period longer than 11.257 days per year can reduce air pollution based on this result and the previous finding of Chauhan and Singh [58] that a lockdown imposed to suspend the operation of businesses temporarily and small-scale industries lowered the volume of traffic and transportation and consequently led to decreased ambient PM2.5 concentrations.

Apart from examining the relationships of lockdowns with the three dependent variables, we also considered the causal links among GDP, PM2.5, and COVID-19 infections in this study. We found a nonlinear relationship in each pair of variables. The results in Table 6 for the ln*GDP* equation reveal that ln*PM* and ln*COVID* levels beyond their kink points at 3.350 and 7.403 are significantly correlated positively and negatively, respectively, with the ln*GDP* level. Specifically, the PM2.5 concentrations above the kink point or concentrations higher than the exp (3.350) = 28.502 µg/m³ level on the average per day vary positively with the GDP levels. This is to say the increasing energy consumption in the urban and industrial sectors of an active economy will go hand in hand with the worsening air quality, as suggested by the results of a study by Rahman, Saidi, and Mbarek [59]. Additionally, a 1% increase in the number of COVID-19 infections after the kink point or more than exp (7.403) = 1640.9 or approximately 1641 cases resulted in a GDP decrease by 0.282%. Furthermore, results in the ln*PM* equation show the positive relationship between air pollution and GDP both for the interval before and that after the kink point, but virtually no direct connection at all between PM2.5 concentrations and the number of COVID-19 infections. This study found a positive association between economic advancement and the COVID-19 spread. The more the economy is developed, the larger the number of coronavirus infections in 2020, as evident in the EU countries, the USA, Japan, and South Korea. Finally, among the control variables, only the COVID-19 recovery index (ln*Index*) and foreign direct investment (FDI) in the first equation have a significant positive relationship with GDP. Our result is consistent with Chaudhry et al. [60], who showed that the increase in patient recovery rates could decrease the vulnerability to biological threats in the country.

Table 7 presents the calculated indirect impacts of lockdowns in one country on the economy, environment, and COVID-19 spread in its neighboring countries. Similarly to the direct impacts, the indirect impacts of lockdowns in one country on the economy and COVID-19 spread in its neighboring countries are quite weak. We observe that the coefficient ln*Lock* > 2.421 is −0.019, at the significance level of 5%; this means lockdowns beyond the kink point (ln*Lock* > 2.421) or exp (2.421) = 11.257 days per year in one country will reduce PM2.5 concentrations in their neighboring countries, and the more lockdown days in a country, the better the air quality in countries nearby.

Then, let us consider the results of the control and endogenous variables from the ln*GDP* equation. We find that there exist significant adverse spillover effects of ln*PM*, ln*Pop*, and ln*r*, from one country to its neighboring countries’ economies. The indirect effects of PM2.5, population density, and interest rate on the neighboring countries’ economies are −0.633, −0.294, and −0.188, respectively. In the ln*PM* equation, one can see the significant impact of ln*GDP* and ln*Open* on ln*PM*. While the trade openness (ln*Open*) contributes a negative impact on the PM2.5 concentration in the neighboring countries, ln*GDP* in one country was detected to have a positive spillover relation. These results show that the increase in trade openness can potentially block the PM2.5 in neighboring countries. The results consistently indicate that trade’s environmental benefits for neighboring countries are found in the processing trade, which is handled by the local country [61]. Finally, in the ln*COVID* equation, only ln*GDP >* 10.301 was found to significantly affect the number of COVID-19 infections in the neighboring countries. 

## 5. Discussion

A significant relationship between lockdowns and the degree of disease spread was found in our study, which is quite consistent with Ghosal, Bhattacharyya, and Majumder [2], who found that lockdowns could suppress the global COVID-19 spread by 61%. However, Farsalinos et al. [32] provided a different view, stating that lockdowns might work well only in the short term by delaying the possibility of virus transmission, but are not a long-lasting solution to suppressing the number of infections and fatalities. Therefore, we consider our findings and conclusions reasonable because the COVID-19 pandemic has remained prevalent up to the present, with a tendency to grow more serious toward the end of 2022 despite restriction measures. With the constant increase and periodical surge in the number of global COVID-19 infections, lockdowns might not be a promising strategy to reduce the number of coronavirus patients. Coccia [15] also confirmed the insignificant effect of lockdowns on COVID-19 spread, as there has not been much difference in the average confirmed cases of countries with short and long periods of lockdowns. The possible reasons are the shortage of healthcare expenditure and an older structure of the population. Indeed, the decision on the short or long duration of a lockdown does not depend on the number of confirmed cases alone, but also depends on the healthcare expenditure and the older population. Many countries with lower healthcare expenditure and older populations have been forced to apply a longer duration of national lockdowns, affording the healthcare system more time to expand and respond to this emergency. Furthermore, apart from examining the relationship of lockdowns with the three dependent variables, we also considered the causal links between the economy, environment, and COVID-19 infections. Our results confirm the nonlinear relationships among them, except for the causal links from the environment to COVID-19 infections. The linear effect of environment on COVID-19 infections is not consistent with the theory of the environmental Kuznets hypothesis, as we did not find the inverted U-shaped relationship between environmental degradation and the country’s income [36,37]. This implies that a sustained rise in income via the expansion of economic activities undermines the state of the environment by generating more pollution [62]. Another reason for obtaining this result is that our data are based on 2020. Thus, the short time span was not long enough to capture the inverted U-shaped relationship. Similar empirical results were also confirmed in the studies by Adu and Denkyirah [63] and N’zué [64]. For the nonlinear nexus between COVID-19 and the economy, our results reveal fresh evidence that the negative impact of COVID-19 spread on the economy becomes more prominent when the number of COVID-19 cases is above 1641. This result is reasonable, as the larger number of COVID-19 cases is associated with a higher degree of anxiety and stress in society and results in the slowdown of economic and social activities. 

Moreover, we also provide evidence of the indirect impacts of lockdowns in one country on the economy, environment, and COVID-19 spread in its neighboring countries. The results can be summarized for the three domains of interest.

1. The higher PM2.5 concentration, greater population density, and higher interest rate of one country will simultaneously create a negative impact on the GDP of its neighboring countries. However, a lockdown in one country was found to have no indirect spatial spillover effects on the neighboring countries.
2. We found that the durations of lockdowns above 11.257 days per year in one country will reduce PM2.5 concentrations in the neighboring countries, and the more the number of lockdown days in a country, the better the air quality in countries nearby. Moreover, while economic development in one country was detected to have a positive spillover relation with PM2.5 concentrations in the neighboring countries, the greater trade openness of a country was found to also contribute to the poorer air quality of adjacent countries.
3. Countries with a GDP per capita of more than USD 29,762.37 per year were shown to result in more cases of coronavirus infections in the close-by countries. This might reflect the fact that economic activities in the high-income economies (compared to Thailand, which has a GDP per capita of USD 5500–6000 per year) continued as in times of normalcy or with normal international labor and human movement during the COVID-19 pandemic, thus resulting in a high spatial spillover of disease spread [33]. Consequently, it might be reasonable to state that there is a trade-off between maintaining the status of a high-income economy and the larger number of COVID-19 infections due to both internal diffusion and cross-border transmission during this global pandemic.

## 6. Conclusions

The conviction that lockdowns are crucial for controlling COVID-19 spread has led many countries to implement this restriction measure. However, the lockdown policies have been shown to impose enormous economic costs on local wealth due to the limitation of economic activities, while in aggregate, causing global economic decline. On the other hand, a lockdown to restrict economic activities and people’s movement turned out to be environmentally beneficial due to lowered private and industrial fuel and energy consumption, particularly for transportation, which is the main source of air pollutant emissions. Therefore, it is possible to have a trade-off between positive and negative outcomes of the lockdown imposition. This research sought to assess the impacts of lockdown on three domains, namely the economy, environment, and COVID-19 spread, using the cross-sectional data of 158 countries around the world in 2020 for the spatial nonlinear econometric analysis by the proposed simultaneous spatial kink equations model. 

The first part of the study dealt with the selection and validation of the optimal model for fitting to the available data sets. Because it was found that some variables have spatial autocorrelation and some have nonlinear features, the proposed model was thus suitable for our data sets. The findings from the kink effect test revealed the significant nonlinear impacts of lockdowns on the economy and environment, while the results of a lockdown did not have any nonlinear effects on COVID-19 confirmed cases, and the model estimation also revealed that lockdowns could not significantly help curb COVID-19 spread. Our results may be contradictory to some previous results of Biswaranjan [29], Rashed et al. [30], Ghosal, Bhattacharyya, and Majumder [2], Atalan [1], and Caselli, Grigoli, and Sandri [28]. The possible reasons for these results can be explained as follows: (1) Because our analysis focused on the global scale (158 countries), the impact of lockdowns on COVID-19 spread can be ambiguous. (2) The COVID-19 crisis is quite new; thus, an appropriate lockdown policy in 2020 may not fit well enough to solve this recent pandemic. Additionally, the unintended consequences of COVID-19, such as those on society, the economy, and the environment, may limit the capability of lockdowns [32]. 

However, this study found that the length of lockdown days is important as the lockdown period of less than 31 days in a year will impose lower economic costs than otherwise. Moreover, the limitation of economic activities was detected to significantly help suppress air pollution, thus supporting the postulation that anthropogenic activities are the primary source of air pollutant emissions and environmental degradation. Finally, among the exogenous variables in the model, the COVID-19 Recovery Index as the control variable can explain better than the lockdown variable the reduction in the number of COVID-19 infections. 

## 7. Policy Recommendations

1. To avoid economic damage, governments should impose a short-duration lockdown (no more than 31 days per year). However, the authorities need to consider the trade-off between economic loss and environmental benefit because more lockdown days can suppress the COVID-19-related panic and fatalities while causing minimum impact on the daily life of people in the society and because the environmental improvement can be pursued as the long-term goal instead. As the present study showed that lockdowns could not suppress the disease spread partly due to ineffective implementation in some countries, governments wishing to control the situation effectively might have to adopt more rigorous restriction measures for a shorter duration or use the complete lockdown alternative once imposed in Wuhan, China, while expediting the vaccination processes for the population.
2. As evident from the spatial spillover study that lockdowns of more than 31.218 days per year in one country could improve air quality in the nearby countries, lockdowns or limitations on economic activities can be strategically employed by policymakers to rapidly and enormously reduce problems related to air pollution. However, the economic loss has to be taken into account in this connection by evaluating the non-monetary environmental benefits and whether the gain can compensate for the losses from shutting down some economic activities.

Despite the above significant academic and policy implications concerning the impact of lockdowns on the economy, environment, and COVID-19 spread, some limitations remain in this study for future research. First, the dataset was collected covering 2020. Thus, the results may not be pertinent to 2021 and 2022, when there emerged some different characteristics, situations, and lockdown policies. Second, we investigated the effects of total lockdown days in 2020, and it should be noted. However, there are conflicting statements regarding the measures used in lockdown policies by countries. Thus, further study may consider the panel of spatial econometric models and include the lockdown characteristics of each country. Third, this study used the total number of days in a lockdown. However, given the incubation period of the virus, there could be different effects from longer consecutive lockdown periods than from the same number of days imposed as lockdowns in a non-consecutive pattern. Using the maximum number of consecutive days of lockdown instead of the total as a comparison can provide a new perspective to explore the mechanisms behind the effects. Furthermore, it will be interesting to investigate the nonlinear and spatial impacts of the COVID-19-induced lockdowns on the economy, environment, and COVID-19 spread in each sub-region. This will enable us to understand the insightful spatial spillover impacts of the COVID-19-induced lockdowns on the economy, environment, and COVID-19 spread. In doing so, it is suggested to increase the number of observations in the panel data. 

## Figures and Tables

**Table 1:** Variable description and descriptive statistics.

Variable	Min	Max	Mean	SD	Description	Source
$\ln GDP_{i}$	6.633	11.678	9.455	1.135	Gross Domestic Product per capita (current USD)	https://m.statisticstimes.com/economy/countries-by-projected-gdp-capita
$\ln PM_{i}$	2.292	4.593	3.691	0.506	Average PM2.5 concentration (µg/m³)	https://statisticstimes.com/economy
$\ln Pop_{i}$	0.746	9.030	4.323	1.364	Population density (people per km^2^)	https://knoema.com
$\ln COVID_{i}$	4.158	11.978	9.541	1.805	The number of COVID-19 confirmed cases (ratio with population). This variable can be viewed as COVID-19 incidence rate.	https://www.worldometers.info/coronavirus/
$\ln Temp_{i}$	−0.400	3.361	2.772	0.713	Average temperature (Celsius)	https://www.weather-atlas.com/
$\ln Lock_{i}$	0.000	5.192	3.700	0.913	Number of days in lockdown per year (Day)	https://graphics.reuters.com/world-coronavirus
$\ln Index_{i}$	3.060	4.542	3.948	0.269	COVID-19 Recovery Index, which measures country’s success in treating patients that have been diagnosed COVID-19 positive	Global Infection Trend—Fu
$\ln Open_{i}$	−4.605	5.501	−0.879	1.630	Trade openness (Ratio with GDP)	https://countryeconomy.com/trade
$\ln r_{i}$	−2.302	4.071	0.964	1.270	Real interest rate (%)	https://tradingeconomics.com/country-list/interest-rate
$\ln FDI_{i}$	−1.840	18.757	6.512	3.108	Foreign direct investment (Million USD)	https://tradingeconomics.com/country-list/foreign-direct-investment

Note: The data is accessed on 9 March 2022.

**Table 2:** Moran’s I values of explanatory variables.

Region	$\ln Lock_{i}$	$\ln PM_{i}$	$\ln COVID_{i}$	$\ln GDP_{i}$	$\ln Pop_{i}$	$\ln Index_{i}$	$\ln Open_{i}$	$\ln FDI_{i}$	$\ln r_{i}$	$\ln Temp_{i}$
World	0.155 ***	0.515 ***	0.578 ***	0.664 ***	0.154 ***	0.180 ***	0.071 **	0.052 *	0.352 ***	0.733 ***
Asia	0.335 ***	0.674 ***	0.335 ***	0.345 ***	0.154 ***	0.180 ***	0.060 *	0.053 *	0.290 ***	0.567 ***
Europe	0.239 **	0.124 ***	0.564 **	0.782 ***	0.201 **	0.203 ***	0.092 *	0.033 *	0.402 ***	0.902 ***
Africa	0.024 **	0.503 **	0.332 ***	0.502 ***	0.029 *	0.120 ***	0.022 *	0.039 *	0.103 ***	0.804 ***
America	0.226 *	0.402 ***	0.302 ***	0.702 ***	0.334 ***	0.048 *	0.089 *	0.060 *	0.420 ****	0.702 ***

Note: “*”, “**”, and “***” denote significance at the 10%, 5%, and 1% levels, respectively.

**Table 3:** The LR test results and the estimated values of kink point.

Dependent Variables	$\ln Lock_{i}$	$\ln PM_{i}$	$\ln COVID_{i}$	$\ln GDP_{i}$	$\ln Pop_{i}$	$\ln Index_{i}$	$\ln Open_{i}$	$\ln FDI_{i}$	$\ln r_{i}$	$\ln Temp_{i}$
	The kink effect test for the spatial lag model									
$\ln GDP_{i}$ Kink point	5.562 *** 3.406	5.198 *** 3.362	3.628 ** 7.399		4.636 ** 3.678	5.237 *** 3.678	0.407	0.963	0.385	
$\ln PM_{i}$ Kink point	4.210 * 2.309		5.342 *** 8.344	6.297 *** 8.369	0.174		0.069			0.145
$\ln COVID_{i}$ Kink point	0.085	0.332		8.594 ** 10.297	0.276	5.227 *** 3.882				
	The kink effect test for the spatial error model									
$\ln GDP_{i}$ Kink point	6.221 *** 3.291	5.983 *** 3.401	3.239 ** 7.219		5.829 *** 3.709	4.294 ** 3.590	0.529	0.920	0.621	
$\ln PM_{i}$ Kink point	4.331 ** 0.2345		4.990 ** 7.301	5.173 *** 5.591	0.290		0.301			0.157
$\ln COVID_{i}$ Kink point	0.086	0.573		8.501 *** 10.256	0.301	5.892 *** 3.903				
	The kink effect test for the spatial Durbin model									
$\ln GDP_{i}$ Kink point	6.192 *** 3.441	5.562 ** 3.350	4.902 ** 7.403		6.093 ** 3.667	5.892 *** 3.692	0.302	0.599	0.291	
$\ln PM_{i}$ Kink point	4.601 ** 0.2421		6.092 *** 8.356	5.688 *** 8.401	0.409		0.321			0.209
$\ln COVID_{i}$ Kink point	0.107	0.733		7.993 *** 10.301	0.331	5.236 *** 3.892				

Notes: “**” and “***” denote significance at the 5%, and 1% levels, respectively, and the number beneath the significant LR corresponds to the estimated kink point value. All independent variables were tested for the kink effect, but the discussion will be made only on those tested to have a kink effect.

**Table 4:** Loglikelihood and BIC values of candidate econometric models.

Linear	Spatial AR	Spatial Error	Spatial Durbin	SLE
Loglikelihood	−463.894	−431.395	−406.816	−475.093
BIC	1018.685	953.687	904.529	1041.083
Nonlinear	Spatial AR	Spatial Error	Spatial Durbin	SKE
Loglikelihood	−402.232	−329.748	**−301.887**	−421.020
BIC	986.259	841.291	**831.018**	1018.785

Note: Bolded numbers correspond to the highest Loglikelihood value and the lowest BIC value.

**Table 5:** Parameter estimates from the simultaneous spatial Durbin kink equation model.

lnGDP		lnPM		lnCOVID	
$\beta_{0}$	6.095 *** (0.756)	$\beta_{0}$	1.749 *** (0.312)	$\beta_{0}$	5.827 *** (1.185)
$\beta_{\ln Lock}^{+}$	−0.596 ** (0.098)	$\beta_{\ln Lock}^{+}$	−0.013 ** (0.005)	$\beta_{\ln Lock}^{}$	−0.101 (0.053)
$\beta_{\ln Lock}^{-}$	−0.203 *** (0.068)	$\beta_{\ln Lock}^{-}$	0.022 (0.019)	$\beta_{\ln GDP}^{+}$	0.685 *** (0.161)
$\beta_{\ln PM_{i}}^{+}$	0.802 *** (0.165)	$\beta_{\ln COVID}^{+}$	0.005 (0.025)	$\beta_{\ln GDP}^{-}$	1.371 *** (0.356)
$\beta_{\ln PM}^{-}$	0.047 (0.262)	$\beta_{\ln COVID}^{-}$	0.006 (0.057)	$\beta_{\ln PM}$	0.021 (0.022)
$\beta_{\ln COVID}^{+}$	−0.278 *** (0.043)	$\beta_{\ln GDP}^{+}$	0.445 *** (0.039)	$\beta_{\ln Index}^{+}$	−1.917 *** (0.568)
$\beta_{\ln COVID}^{-}$	−0.057 (0.101)	$\beta_{\ln GDP}^{-}$	0.370 *** (0.091)	$\beta_{\ln Index}^{-}$	−1.038 (1.126)
$\beta_{\ln Pop}^{+}$	0.044 (0.046)	$\beta_{\ln Pop}^{}$	−0.003 (0.018)	$\beta_{\ln Pop}^{}$	0.056 *** (0.010)
$\beta_{\ln Pop}^{-}$	−0.128 (0.079)	$\beta_{\ln Open}^{}$	−0.015 (0.014)	$\beta_{W\ln Lock}^{}$	−0.209 * (0.105)
$\beta_{\ln Index}^{+}$	0.647 ** (0.254)	$\beta_{\ln Temp}^{}$	−0.070 (0.069)	$\beta_{W\ln GDP}^{+}$	0.195 (0.218)
$\beta_{\ln Index}^{-}$	0.109 (0.504)	$\beta_{W\ln Lock}^{+}$	−0.180* (0.102)	$\beta_{W\ln GDP}^{-}$	−0.217 (0.609)
$\beta_{\ln Open}^{}$	−0.030 (0.027)	$\beta_{W\ln Lock}^{-}$	−0.010 (0.062)	$\beta_{W\ln PM}$	0.002 (0.003)
$\beta_{\ln FDI}^{}$	0.047 *** (0.016)	$\beta_{W\ln COVID}^{+}$	−0.005 (0.038)	$\beta_{W\ln Index}^{+}$	0.319 (0.985)
$\beta_{\ln r}^{}$	−0.067 (0.041)	$\beta_{W\ln COVID}^{-}$	−0.073 (0.110)	$\beta_{W\ln Index}^{-}$	0.473 (1.823)
$\beta_{W\ln Lock}^{+}$	−0.242 ** (0.118)	$\beta_{W\ln GDP}^{+}$	−0.140 (0.067)	$\beta_{W\ln Pop}^{}$	−0.069 (0.116)
$\beta_{W\ln Lock}^{-}$	0.225 (0.170)	$\beta_{W\ln GDP}^{-}$	0.193 ** (0.147)		
$\beta_{W\ln PM_{i}}^{+}$	−0.691 *** (0.235)	$\beta_{W\ln Pop}^{}$	0.051 (0.033)		
$\beta_{W\ln PM}^{-}$	0.185 (0.377)	$\beta_{W\ln Trade}^{}$	−0.038 (0.024)		
$\beta_{W\ln COVID}^{+}$	0.008 (0.065)	$\beta_{W\ln Temp}^{}$	0.052 (0.085)		
$\beta_{W\ln COVID}^{-}$	−0.009 (0.199)				
$\beta_{W\ln Pop}^{+}$	0.101 (0.081)				
$\beta_{W\ln Pop}^{-}$	−0.216 (0.151)				
$\beta_{W\ln Index}^{+}$	0.203 (0.457)				
$\beta_{W\ln Index}^{-}$	−0.195 (0.874)				
$\beta_{W\ln Open_{i}}^{}$	0.038 (0.048)				
$\beta_{W\ln FDI}^{}$	−0.022 (0.035)				
$\beta_{W\ln r}^{}$	−0.141 * (0.072)				
ρ	0.210 **	ρ	0.359 ***	ρ	0.390 ***

Notes: “*”, “**”, and “***” denote significance at the 10%, 5%, and 1% levels, respectively, and values within the () are the standard error.

**Table 6:** Analysis of lockdown’s direct effects.

lnGDP		lnPM		lnCOVID	
$Direct_{\ln Lock}^{+}$	−0.312 ** (0.100)	$Direct_{\ln Lock}^{+}$	−0.013 *** (0.002)	$Direct_{\ln Lock}^{}$	−0.080 * (0.037)
$Direct_{\ln Lock}^{-}$	−0.193 *** (0.068)	$Direct_{\ln Lock}^{-}$	0.024 (0.042)	$Direct_{\ln GDP}^{+}$	0.740 ** (0.158)
$Direct_{\ln PM_{i}}^{+}$	0.773 *** (0.165)	$Direct_{\ln COVID}^{+}$	0.005 (0.026)	$Direct_{\ln GDP}^{-}$	1.409 *** (0.367)
$Direct_{\ln PM}^{-}$	0.058 (0.260)	$Direct_{\ln COVID}^{-}$	0.001 (0.060)	$Direct_{\ln PM}$	−0.099 (0.094)
$Direct_{\ln COVID}^{+}$	−0.282 *** (0.042)	$Direct_{\ln GDP}^{+}$	0.447*** (0.038)	$Direct_{\ln Index}^{+}$	−1.968 *** (0.607)
$Direct_{\ln COVID}^{-}$	−0.058 (0.104)	$Direct_{\ln GDP}^{-}$	0.363*** (0.094)	$Direct_{\ln Index}^{-}$	−1.030 (1.153)
$Direct_{\ln Pop}^{+}$	0.050 (0.047)	$Direct_{\ln Pop}^{}$	−0.001 (0.020)	$Direct_{\ln Pop}^{}$	0.050 *** (0.013)
$Direct_{\ln Pop}^{-}$	−0.141 (0.080)	$Direct_{\ln Open}^{}$	−0.020 (0.016)		
$Direct_{\ln Index}^{+}$	0.666 ** (0.262)	$Direct_{\ln Temp}^{}$	−0.067 (0.065)		
$Direct_{\ln Index}^{-}$	0.121 (0.528)				
$Direct_{\ln Open}^{}$	−0.028 (0.028)				
$Direct_{\ln FDI}^{}$	0.046 *** (0.017)				
$Direct_{\ln r}^{}$	−0.076 (0.040)				

Notes: “*”, “**” and “***” denote significance at the 10%, 5%, and 1% levels, respectively; values within the () are the standard error.

**Table 7:** Analysis of lockdown’s indirect effects.

lnGDP		lnPM		lnCOVID	
$Indirect_{\ln Lock}^{+}$	0.344 (0.224)	$Indirect_{\ln Lock}^{+}$	−0.019 ** (0.154)	$Indirect_{\ln Lock}^{}$	−0.258 (0.130)
$Indirect_{\ln Lock}^{-}$	0.221 (0.134)	$Indirect_{\ln Lock}^{-}$	−0.026 (0.090)	$Indirect_{\ln GDP}^{+}$	0.703 *** (0.271)
$Indirect_{\ln PM_{i}}^{+}$	−0.633 ** (0.284)	$Indirect_{\ln COVID}^{+}$	−0.004 (0.052)	$Indirect_{\ln GDP}^{-}$	0.483 (0.946)
$Indirect_{\ln PM}^{-}$	0.236 (0.470)	$Indirect_{\ln COVID}^{-}$	−0.104 (0.175)	$Indirect_{\ln PM}$	−0.083 (0.056)
$Indirect_{\ln COVID}^{+}$	0.080 (0.065)	$Indirect_{\ln GDP}^{+}$	0.029 (0.073)	$Indirect_{\ln Index}^{-}$	0.103 (2.850)
$Indirect_{\ln COVID}^{-}$	−0.026 (0.246)	$Indirect_{\ln GDP}^{-}$	0.088 ** (0.212)	$Indirect_{\ln Pop}^{}$	−0.072 (0.173)
$Indirect_{\ln Pop}^{+}$	0.134 (0.099)	$Indirect_{\ln Pop}^{}$	0.072 (0.050)	$Indirect_{\ln Index}^{+}$	−0.651 (1.502)
$Indirect_{\ln Pop}^{-}$	−0.294 * (0.176)	$Indirect_{\ln Open}^{}$	−0.065 * (0.038)		
$Indirect_{\ln Index}^{+}$	0.411 (0.572)	$Indirect_{\ln Temp}^{}$	0.039 (0.102)		
$Indirect_{\ln Index}^{-}$	−0.264 (1.123)				
$Indirect_{\ln Open}^{}$	0.038 (0.062)				
$Indirect_{\ln FDI}^{}$	−0.014 (0.045)				
$Indirect_{\ln r}^{}$	−0.188 ** (0.085)				

Notes: “*”, “**,” and “***” denote significance at the 10%, 5%, and 1% levels, respectively; values within the () are the standard error.

## Data Availability

The datasets analyzed during the current study are available at Thomson Reuter DataStream.

## Appendix A

**Table A1:** List of the countries considered in this study.

Afghanistan	Brunei	Guinea	India	Malawi	Oman	Somalia	Venezuela
Albania	Bulgaria	Eritrea	Indonesia	Malaysia	Pakistan	South Africa	Vietnam
Algeria	Burkina Faso	Estonia	Iran	Maldives	Panama	Spain	Zambia
Angola	Burundi	Eswatini	Iraq	Mali	Papua New Guinea	Sri Lanka	Zimbabwe
Antigua and Barbuda	Cambodia	Ethiopia	Ireland	Malta	Paraguay	Sudan	
Argentina	Cameroon	Fiji	Israel	Mauritania	Peru	Suriname	
Armenia	Canada	Finland	Italy	Mauritius	Philippines	Sweden	
Australia	Chile	France	Jamaica	Mexico	Bhutan	Switzerland	
Austria	China	Gabon	Japan	Mongolia	Poland	Tajikistan	
Azerbaijan	Colombia	Gambia	Jordan	Montenegro	Portugal	Thailand	
Bahamas	Comoros	Georgia	Kazakhstan	Morocco	Qatar	Timor	
Bahrain	Costa Rica	Germany	Kenya	Mozambique	Romania	Togo	
Bangladesh	Croatia	Ghana	Kuwait	Myanmar	Russian	Trinidad	
Barbados	Cuba	Greece	Kyrgyzstan	Namibia	Sao Tome and Principe	Tunisia	
Belarus	Cyprus	Guatemala	Lao	Nepal	Saudi Arabia	Turkey	
Belgium	Czechia	Guinea	Latvia	Netherlands	Senegal	Uganda	
Belize	Denmark	Guinea-Bissau	Lebanon	New Zealand	Serbia	Ukraine	
Benin	Djibouti	Guyana	Lesotho	Nicaragua	Seychelles	UAE	
Bhutan	Dominican	Haiti	Liberia	Niger	Sierra Leone	UK	
Bolivia	Ecuador	Honduras	Lithuania	Nigeria	Singapore	USA	
Botswana	Egypt	Hungary	Luxembourg	North Macedonia	Slovakia	Uruguay	
Brazil	El Salvador	Iceland	Madagascar	Norway	Slovenia	Uzbekistan	
